# Defining Sub-Saharan Africa’s Health Workforce Needs: Going Forwards Quickly Into the Past: Comment on "Non-physician Clinicians in Sub-Saharan Africa and the Evolving Role of Physicians"

**DOI:** 10.15171/ijhpm.2016.100

**Published:** 2016-08-15

**Authors:** E. Oluwabunmi Olapade-Olaopa, Nelson K. Sewankambo, Jehu E. Iputo

**Affiliations:** ^1^College of Medicine, University of Ibadan, Ibadan, Nigeria.; ^2^College of Health Sciences, Makarere University, Kampala, Uganda.; ^3^Department of Medical Education, Walter Sisulu University, Mthatha, South Africa.

**Keywords:** Medical Education, Human Resources for Health, African Health Systems, Non-physician Clinicians (NPCs), Task-Shifting, Health System Reform

## Abstract

Recent proposals for re-defining the roles Africa’s health workforce are a continuation of the discussions that have been held since colonial times. The proposals have centred on basing the continent’s healthcare delivery on non-physician clinicians (NPCs) who can be quickly trained and widely distributed to treat majority of the common diseases. Whilst seemingly logical, the success of these proposals will depend on the development of clearly defined professional duties for each cadre of healthcare workers (HCW) taking the peculiarities of each country into consideration. As such the continent-wide efforts aimed at health-professional curriculum reforms, more effective utilisation of task-shifting as well as the intra – and inter-disciplinary collaborations must be encouraged. Since physicians play a major role in the training mentoring and supervision of physician and non-physician health-workers alike, the maintenance of the standards of university medical education is central to the success of all health system models. It must also be recognized that, efforts at improving Africa’s health systems can only succeed if the necessary socio-economic, educational, and technological infrastructure are in place.

"*Whatever device is created for dealing with the sheer volume of health work (in Africa), it must not be one which compromises the standards of university medical school education*."

A. Brown. ‘Medical Education in Nigeria.’ (1963)

The debate on the most appropriate structure for Africa’s health-workforce in general, and its physicians in particular, has been generated by the mismatch of the volume of disease and the number of available trained healthcare workers (HCW).^1^ This mismatch has been further complicated in recent times by the increase in, aging and mobility of the population, the recurring epidemics of infectious diseases, and the continuing brain drain, all of which further amplify the continent’s human resource for health (HRH) shortage.^2,3^

Recent proposals for solving Africa’s health workforce challenges^4,5^ are a continuation of the discussions that have been held since the need for a formal health system was identified in colonial times.^6^ Several of the proposals have focused on task-shifting to be facilitated by the large-scale production of non-physician clinicians (NPCs) who can be quickly trained and distributed widely to treat majority of the common diseases, and by re-defining the roles of the various cadres of HCW.^4,5,7^ Others are directed at scaling-up the quantity and quality of health-professionals as well as increasing the retention of medical doctors being produced within the sub-continent as a longer term solution.^8,9^ These later set of objectives are being achieved through targeted admissions.^10^ curricular reforms,^11^ continuing professional development,^12^ and inter-professional educations.^13^

This article comments on one such proposal.^4^

## The Prevailing Challenges of Africa’s Health Systems

The inadequate number and mal-distribution of HCWs has meant sub-Saharan Africa (SSA’s) health workforce is unable to respond to its current health challenges. This is because the models of health systems utilized so far in the sub-continent have been mainly adopted from foreign countries with limited integration with the culture and customs of the host countries. Also, most SSA health systems have developed largely without the concomitant development of the robust administrative policies and supporting social, economic, and technological infrastructure required for their successful function.^14^

Financing the cost of increasing the number of HCW and the facilities available for their use in SSA is well-recognized health systems challenge since most countries have inadequate health and education budgets.^15^ Further, the cost-recovery policies of health institutions in the sub-continent pose a formidable barrier for many patients who are unable to pay the high fees.^16^

Another major challenge to Africa’s health system is the lack of definition of the role of each cadre of HCW (physician and non-physician alike) and their relationships to each other. This situation has been further complicated by the near total absence of regulatory structures to monitor NPCs in most SSA countries.^17-21^ Furthermore, instances of NPCs delivering care outside their scope or without supervision have been reported in developing countries and are a major concern to other HCW especially physicians.^22-24^ Un-coordinated expansion of task-shifting also has the added potential of paradoxically diluting efforts to improve the training and quality of physicians. This is because this may reduce the patient load available for training medical students while increasing demands on physicians now saddled with additional responsibilities of supervision and training of non-physician HCWs and thus reduce important direct physician patient care in practice. These deficiencies have enshrined professional rivalries resulting in persistent struggles for practice territories and hierarchy, and inevitably incessant health sector crises.^24^

The non-standardisation of NPC training and practice in Africa, unlike what obtains in developed countries (notably the United States), poses a major challenge to its health systems.^25,26^ This is because NPC training in Africa is largely informal and sometimes personal,^27^ whilst the few vocational schools that offer formal training prescribe different lengths of training.^18,19,26^ Indeed, most countries utilising NPCs are yet to formalize their training health sector role, career path, and/or relationship relative to other established cadre of health-workers.^20-22^ This may eventually lead to frustration on the part of the NPCs, and suspicion and refusal of acceptance by the other cadres especially the physicians making successful supervision and training of NPCs difficult to replicate.^27^

The brain drain of physicians and nurses has continued unabated in part because majority of governments are yet to address the local workforce challenges.^28^ Skill mix imbalances, urban-rural distribution imbalances, poor employment and career prospects, and poor working conditions further contribute to workforce challenges in many developing countries and may contribute to perpetuating the brain drain.^26,29^

## Remembering a Solution From the Past

It is particularly important that SSA’s health systems maximize the available HRH for the effective and efficient health coverage of its people and task-shifting (including the use of NPCs as appropriate) is a major component. However, defining the standards of training and practice for SSA’s physicians and non-physician health-workers alike should be the first consideration. This will involve the development of clearly defined and achievable professional duties for each cadre of HCW taking the peculiarities of the health-workforce, culture and infrastructure of individual countries into consideration. In this manner, disputes over professional territories, career progression, and hierarchy which are central to health sector conflicts should be minimized.

The need for task-shifting as proposed above was first clearly stated by Alexander Brown in 1963 when he asserted that it would be necessary to transfer aspects of medicine to well-trained non-medical personnel so as to relieve the physician of a significant proportion of clinical work.^30^ He, however, emphasized the need to maintain the standards of physician training as, in this situation, this cadre of HCW would remain the back-bone of the health-workforce and would now be called upon mostly for complex cases.

Alexander Brown’s proposal encompasses the need for re-defining the role of all HCW periodically to make them fit-for-practice in their community. This will be achieved through continuous curricular reforms which should equip each health-professional with the new skills required for contemporary practice in their health system. In this vein, ongoing medical curricular reforms include the modules that teach the administrative and managerial skills proposed by Eyal et al.^4^ These also encompass inter-professional education modules to foster good relationships between all HCW and enshrine the inter-disciplinary healthcare approach necessary for effective patient care.

Brown’s assertion also provides the framework for defining the roles for physician and other HCW, especially NPCs, and prescribing the relationship between them. Importantly, the relationships will be more easily guided once the competencies; expected professional activities and essential professional duties of the different HCW are developed following on the recent emphasis on a need for same standard for of physicians across the sub-contiment.^31^ Additionally, processes must be established to improve the relationship between physicians and other HCW (especially NPCs) as these are necessary for the effective supervision of non-physician HCW working in the primary healthcare setting.^32^ The suggested revision of medical curricula by Eyal et al, to include tracks for NPCs may encourage the introduction acceptance and survival of NPC training and services in some countries. On the other hand, others may find it more convenient to enable qualified paramedicals to acquire additional clinical skills in order to facilitate task-lshifting.^5,8^

Finally, the culture of networking between SSA’s medical schools initiated in the early 1960s (by the formation of the Association of Medical Schools of Africa)^33^ which improved the standard of health-education in the sub-continent should now be extended to involve the health systems in order to achieve the same with healthcare delivery. Indeed, these collaborations have been strengthened by the numerous national and international health-systems strengthening projects and have served to sustain the gains of these initiatives.^5,34^

## Conclusion

The current efforts to re-define the roles of HCW in SSA present an opportunity for SSA to develop health systems that are culturally and socially acceptable, and thus sustainable. As such the continent-wide efforts aimed at health-professional curriculum reforms, wide-spread utilisation of task-shifting and NPCs and the expansion of intra- and inter-professional collaborations must be encouraged. If well-harnessed, the new methods of delivering healthcare services equitably to Africa’s populations may result in a paradigm shift leading to better health statistics and outcomes. The maintenance of the standards of university medical education is however, central to the success of whatever model of health system is developed or chosen. Despite this, it must be remembered that efforts at restructuring Africa’s health-workforce can only succeed if there are concomitant efforts at improving the socio-economic, educational, and technological infrastructure of the individual countries as these are the pillars on which health systems are built.

## Acknowledgements

The authors acknowledge the editorial contributions of Dr. A. T Orunmuyi in preparing this manuscript for publication.

## Ethical issues

Not applicable.

## Competing interests

Authors declare that they have no competing interests.

## Authors’ contributions

All authors contributed to the concept, content, and writing of this manuscript.

## Authors’ affiliations

^1^College of Medicine, University of Ibadan, Ibadan, Nigeria. ^2^College of Health Sciences, Makarere University, Kampala, Uganda. ^3^Department of Medical Education, Walter Sisulu University, Mthatha, South Africa.

## References

[1] Eyal E, Cancedda C, Kyamanywa P, Hurst SA (2015). Non-physician clinicians in sub-Saharan Africa and the evolving role of physicians. Int J Health Policy Manag.

[2] Thorp E. Ladder of Bones: The Birth of Modern Nigeria From 1853 to Independence. London; Glasgow Collins; 1966.

[3] World Health Organization (WHO). Tasks shifting: rational redistribution of tasks among health workforce teams: Global recommendations and guidelines. Geneva, Switzerland: WHO; 2008.

[4] Deller B, Tripathi V, Stender S (2015). Task shifting in maternal and newborn healthcare: key components from policy to implementation. Int J Gynaecol Obstet.

[5] Ellard DR, Shemdoe A, Mazuguni F (2016). Can training non-physicians/associate clinicians (NPCs/ACs) in emergency obstetric, neonatal care and clinical leadership make a difference to practice and help towards reductions in maternal and neonatal mortality in rural Tanzania? The ETATMBA project. BMJ Open.

[6] Monekosso GL. A brief history of medical education in Sub-Saharan Africa. Celebrating_accountable_medical_education_in_Africa.pdf. https://monekosso.files.wordpress.com. Accessed June 10, 2016. Published 2010.

[7] Monekosso GL (2014). A brief history of medical education in sub-Sahara Africa. Acad Med.

[8] Church Missionary Society Archive. http://www.ampltd.co.uk/digital_guides/church_missionary_society_archive_general/editorial%20introduction%20by%20rosemary%20keen.aspx.Accessed June 8, 2016.

[9] Melamby K. The birth of Nigeria’s University. London: Methuen; 1958.

[10] Iliffe J. East African Doctors: A History of the Modern Profession. Cambridge: Cambridge University Press; 1998.

[11] Mullan F, Freywot S, Omaswa F (2011). Medical schools in sub-Saharan Africa. Lancet.

[12] Dgedge M, Mendoza A, Necochea E, Bossemeyer D, Rajabo M, Fullerton J (2012). A survey of sub-Saharan African medical schools. Hum Resour Health.

[13] Kiguli-Malwadde E, Olapade-Olaopa EO, Kiguli S (2014). Competency-based medical education in two sub-Saharan African medical schools. Adv Med Educ Pract.

[14] Olaleye DO, Odaibo GN, Carney P (2014). Enhancement of health research capacity in Nigeria through north-south and in-country partnerships. Acad Med.

[15] Cancedda C, Farmer PE, Kerry V (2015). Maximising the impact of training initiatives for health professionals in low-income countries: frameworks, challenges and best practices. PLoS Med.

[16] Olapade-Olaopa EO, Baird S, Kiguli-Malwadde E, Kolars JC (2014). Growing partnerships: leveraging the power of collaboration through the Medical Education Partnership Initiative. Acad Med.

[17] Benavides B, Caffrey M. Incorporating lay human resources to increase accessibility to antiretroviral therapy: a home-based approach in Uganda. Chapel Hill: The capacity project; 2006. http://www.capacityproject.org/images/stories/files/promising_practices_uganda.pdf. Accessed June 8, 2016.

[18] Chu K, Rosseel P, Gielis P, Ford N (2009). Surgical task shifting in sub-Saharan Africa. PLoS Med.

[19] Fulton BD, Scheffler RM, Sparkes SP, Auh EY, Vujicic M, Soucat A (2011). Health workforce skill mix and task shifting in low income countries: a review of recent evidence 2011. Human Resour Health.

[20] Johan du Plessis, Pitcher R (2015). Towards task shifting? A comparison of the accuracy of acute trauma-radiograph reporting by medical officers and senior radiographers in an African hospital. Pan Afr Med J.

[21] Labhardt ND, Balo J, Ndam M, Grimm J, Manga M (2010). Task shifting to non-physician clinicians for integrated management of hypertension and diabetes in rural Cameroon: a programme assessment at two years. BMC Health Serv Res.

[22] Mullan F, Frehywot S (2007). Non-physician clinicians in 47 sub-Saharan African countries. Lancet.

[23] Yaya Bocoum F, Kouanda S, Kouyate B, Hounton S, Adam T (2013). Exploring the effects of task shifting for HIV through a systems thinking lens: the case of Burkina Faso. BMC Public Health.

[24] Lehmann U, Van Damme W, Barten F, Sanders D (2009). Task shifting: the answer to the human resources crisis in Africa?. Hum Resour Health.

[25] Rustagi AS, Manjate RM, Gloyd S (2015). Perspectives of key stakeholders regarding task shifting of care for HIV patients in Mozambique: a qualitative interview-based study with Ministry of Health leaders, clinicians and donors. Hum Resour Health.

[26] Munga MA, Kilima SP, Mutalemwa PP, Kisoka WJ, Malecela MN (2012). Experiences, opportunities and challenges of implementing task shifting in underserved remote stings: the case of Kongwa district, central Tanzania. BMC Int Health Hum Rights.

[27] Dambisya YM, Matinhure S (2012). Policy and pragmatic implications of task shifting in Uganda: a case study. BMC Health Serv Res.

[28] Olapade-Olaopa EO, Sewankambo NK, Iputo J, et al. Consensus on African Physician Competencies. Presentation at: the 2015 MEPI Symposium; July 15, 2015; Harare, Zimbabwe.

[29] Kwizera EN, Iputo JE (2011). Addressing social responsibility in medical education: the African way. Med Teach.

[30] Iputo JE (2008). Faculty of Health Sciences, Walter Sisulu University: training doctors from and for rural South African communities. MEDICC Rev.

[31] Gilbert JH, Yan J, Hoffman SJ (2010). A WHO report: framework for action on inter-professional education and collaborative practice. J Allied Health.

[32] World Health Organization (WHO). Transforming and scaling up health professionals’ education and training. Geneva: WHO; 2013. http://apps.who.int/iris/bitstream/10665/93635/1/9789241506502_eng.pdf. 26042324

[33] American Academy of Physician Assistants (AAPA) website. https://www.aapa.org. Accessed June 6, 2016.

[34] Eric A, Friedman JD. An action plan to prevent brain drain: building equitable health systems in Africa. Boston MA: Physicians for Human Rights; 2004. http://allafrica.com/download/resource/main/main/idatcs/00010242:21e6b22646882263f8b7aa73a71c810c.pdf. Accessed June 6, 2016.

